# A hybrid machine learning-enhanced MCDM model for transport safety engineering

**DOI:** 10.1038/s41598-025-21297-8

**Published:** 2025-10-20

**Authors:** Xingjian Zhang, Haowen Chen, Jingxuan Chen, Hanrui Feng, Mingshuo Liu, Xushuai Zhang, Aaron Kaiqiang Zhou, Ziyan Li, Anqi Song, Ziang Wu, Surui Cheng, Yuhan Gan, Minghe Liu, Junyu Quan, Shuolei Gao, Mingren Zheng, Faan Chen

**Affiliations:** 1 Courant Institute of Mathematical Sciences, New York University, New York, NY 10012 USA; 2 Department of Economics, Boston University, Boston, MA 02215 USA; 3 School of Economics and Management, East China Normal University, Shanghai, 200241 China; 4 Department of Mathematics, University of Chicago, Chicago, IL 60637 USA; 5 Department of Software Engineering, Northeastern University, Shenyang, 110169 Liaoning China; 6 Department of Electrical and Computer Engineering, Boston University, Boston, MA 02215 USA; 7 Schulich School of Business, York University, Toronto, ON M3J 1P3 Canada; 8 College of Radio and Television, Communication University of China Nanjing, Nanjing, 211172 Jiangsu China; 9 School of Computer and Electronic Information, Nanjing Normal University, Nanjing, 210023 Jiangsu China; 10 Tandon School of Engineering, New York University, New York, NY 11201 USA; 11 Stony Brook Institute, Anhui University, Hefei, 230039 Anhui China; 12 School of International Trade and Economics, Central University of Finance and Economics, Beijing, 102206 China; 13 School of Management, University College London, London, E14 5AA UK; 14 Department of Mathematics, University of California at Santa Barbara, Santa Barbara, CA 93106 USA; 15 School of Economics, University of Sydney, Camperdown, NSW 2006 Australia; 16 Tabor Academy, Marion, MA 02738 USA; 17 School of Engineering and Applied Sciences, Harvard University, Cambridge, MA 02138 USA

**Keywords:** Decision reliability, Transport safety engineering, K-means, Machine learning, Graph-based technique, OAS, Engineering, Civil engineering, Scientific data

## Abstract

Delivering reliable decision recommendations and policy inferences is essential for multi-criteria decision-making (MCDM) processes, particularly for transport safety engineering. This study proposes a hybrid machine learning-enhanced MCDM model that integrates distance correlation-based criteria importance through intercriteria correlation (DCRITIC), weighted aggregated sum product assessment (WASPAS), and K-means clustering, referred to as the DCRITIC–WASPAS–K-means model. In particular, we incorporated a machine learning tool (i.e., a graph-based technique) into the model to effectively and robustly select initial centroids. This integration addresses the uncertainty in traditional k-means clustering, which arises from varying initial centroids and its sensitivity to outliers, especially in datasets with noisy or skewed data points, and, more importantly, reduces the number of iterations and runtime cost. This approach improves the robustness and reliability of decision outcomes, thereby supporting more credible and actionable policy interventions. A case study involving transport safety engineering in the Organization of American States (OAS) region validates the model’s practical utility. Comparative analyses demonstrate its superior performance in ensuring consistent decision outputs and communicating policy implications effectively. The proposed framework provides public administrators, policymakers, and government agencies with a reliable, scalable, and data-driven tool for strategic planning and resource allocation in uncertain environments.

## Introduction

Transport safety poses a critical global issue with profound implications for both public health and economic development. Annually, road traffic crashes claim the lives of more than 1.35 million individuals and injure up to 50 million others, positioning road-related incidents among the leading causes of mortality worldwide^1^. Low- and middle-income nations are responsible for nearly 90% of all road traffic fatalities worldwide, with a significant proportion occurring in member states of the Organization of American States (OAS). Dramatically, road traffic injuries in the Americas led to an estimated 150,000 deaths in 2010, ranking as the second most common cause of mortality among individuals aged 15 to 24^2^. This underscores the pressing necessity for ongoing improvement efforts to strengthen the management of transport safety across different geographic contexts. Achieving meaningful management outcomes (i.e., decision conclusions and policy recommendation) depends heavily on multi-criteria decision-making (MCDM), which requires the synthesis of multiple analytical approaches^3^. MCDM model serves as an essential decision-support tool, particularly useful in addressing complex challenges that involve multiple criteria or objectives. It enables decision-makers to identify the optimal solution from a range of alternatives, while managing trade-offs and prioritizing various criteria. Therefore, ensuring its stability, reliability, and efficiency is critical in MCDM applications, especially in areas such as safety engineering.

However, selecting the most appropriate method based on these attributes from the extensive array of options available in MCDM libraries presents a significant challenge for decision-makers^4^. Additionally, MCDM models inherently exhibit sensitivity and uncertainty regarding input data, which necessitates the development of MCDM models that can internally manage these challenges^5–7^. Numerous studies have provided a solid foundation for this research^6,8–13^. Despite this, research gaps persist: (1) Previous methodologies have primarily focused on a single jurisdiction, such as a country, city, or county, overlooking the complexities of transnational diversity among jurisdictions. This limitation diminishes the universality and practical applicability of MCDM in real-world scenarios. (2) Many methods in prior studies focused solely on aggregation (i.e., ranking alternatives) while neglecting grouping, deconstruction, and decomposition, which are essential for enhancing decision-making effectiveness. (3) Most previous methodologies have encountered issues of outcome sensitivity and uncertainty due to variations in input data. This challenge is particularly acute when dealing with small to medium-sized datasets, where the inherent instability of the models becomes more apparent.

To address these gaps, this study developed an advanced and robust MCDM framework that combines distance correlation-based criteria importance through intercriteria correlation (DCRITIC), weighted aggregated sum product assessment (WASPAS), and K-means clustering, termed the DCRITIC–WASPAS–K-means model. The objective is to create a decision tool that encompasses various stages of the MCDM process, including weighting, aggregation, grouping, and decomposition, while ensuring significant efficiency, reliability, and stability. Specifically, we introduced a machine learning algorithm (i.e., a graph-based technique) into the model to enhance its stability and the reliability of its outcomes. This study offers original value and makes significant contributions to both academic research and industry applications.

- First, this research constructs a set of safety performance indicators (SPIs) to assess and guide transport safety efforts, providing a crucial basis for policy and decision-making within the OAS member countries.
- Second, this study addresses limitations inherent in traditional *k*-means clustering by integrating a graph-based machine learning technique. This enhancement effectively selects initial centroids, thus mitigating the uncertainty in traditional *k*-means clustering caused by varying initial centroids and reducing its sensitivity to outliers, especially in datasets with noisy or skewed data points. This methodological refinement significantly strengthens the utility of clustering in transport safety engineering, enabling more consistent group-based assessments and supporting data-informed policy formulation under complex and uncertain conditions.
- Third, the study equips government officials, policymakers, and decision-makers with a dependable decision-support management tool, thereby establishing a solid foundation for directing strategic initiatives and actionable measures concerning transport safety across OAS member countries.

The rest of this paper is organized as follows: section “Literature review” discusses the uncertainties inherent in the MCDM process, explores the applications and limitations of *k*-means clustering, and identifies gaps in the existing research. Section “Methodology” details the SPIs utilized in this study and describes the data sources. Section “Results and discussion” explains the MCDM model developed for this research. Section “Practical implications” details the computational findings and performs a robustness assessment. Section “Concluding remarks” discusses the policy significance of empirical findings. The paper concludes with section “Conclusion”, which offers the final remarks and suggests directions for future research.

## Literature review

### Uncertainty in the MCDM process

In the MCDM process, uncertainty arises from incomplete knowledge or information regarding various factors that greatly influence the quality and reliability of decision outcomes. This uncertainty can originate from multiple sources, including data uncertainty, model uncertainty, decision-maker uncertainty, and environmental uncertainty^6,14–17^.

First, data uncertainty refers to the uncertainties related to the input data used in the decision-making process, such as measurement errors, missing data, and inconsistencies^18^. These uncertainties may affect the input values for criteria and alternative evaluations. Second, model uncertainty arises from the complex mathematical or computational algorithms used in MCDM models, the assumptions within these models, the selection of model parameters, or the accuracy of the model in representing real-world scenarios^14^. Third, decision-maker uncertainty pertains to the subjective judgments and preferences of decision-makers, which can vary due to different risk attitudes or conflicting priorities^19,20^. Additionally, external factors, such as policy changes and technological advancements, introduce uncertainty into the decision-making process, potentially influencing the outcomes or consequences of decision alternatives.

Uncertainty can lead to suboptimal decisions or increased risk exposure if not properly managed in the MCDM process. This may compromise the credibility of decision outcomes and diminish stakeholder confidence in the decision-making processes. Additionally, uncertainty can cause delays or indecision as decision-makers struggle with ambiguous or conflicting information. Addressing uncertainty in the MCDM process is essential for making robust and reliable decisions. Strategies for managing uncertainty include sensitivity analysis^21,22^, scenario analysis^23^, decision-tree analysis^24^, Monte Carlo simulations^4^, and stakeholder engagement to incorporate diverse perspectives and expertise.

### K-means application and limitations

Originally introduced by^25^ and further refined by^26^, K-means clustering is a commonly applied unsupervised learning technique that groups data into clusters according to similarity patterns. Its popularity across various domains is owing to its simplicity, efficiency, and effectiveness in certain scenarios^27^, including customer segmentation^28^, image compression^29^, anomaly detection^30^, document clustering^31^, market basket analysis^32^, recommendation systems^33^, genomic data analysis^34^, network traffic analysis^35^. These applications underscore the versatility of *k*-means clustering across various domains, highlighting its utility in data analysis, pattern recognition, and decision-making processes.

Despite its widespread use, *k*-means clustering exhibits several limitations. First, the performance of the algorithm is highly sensitive to the initial placement of centroids. Depending on the initial positions, the algorithm may converge to different solutions, leading to variability in the results^36^. Second, *k*-means assumes that clusters are spherical and of similar sizes, which may not hold true for real-world data, potentially leading to suboptimal results^37^. Third, outliers can significantly influence the outcomes of *k*-means clustering. Since the algorithm minimizes the sum of squared distances from data points to their respective cluster centroids, outliers can distort cluster boundaries and result in incorrect assignments^38^. Fourth, *k*-means struggles with data that are not linearly separable, as it fails to identify clusters with complex shapes or nonlinear boundaries due to its dependence on Euclidean distances^39^.

Additionally, *k*-means requires the specification of the number of clusters (*k*) beforehand, which can be challenging to determine accurately. An incorrect *k*-value can lead to suboptimal clustering results. Moreover, the algorithm may converge to local optima, especially in high-dimensional spaces or noisy environments^40^. Multiple initializations and averaging results across runs are often necessary to mitigate this issue. Furthermore, *k*-means clustering is sensitive to feature scaling; larger-scale features can dominate the clustering process, leading to biased assignments^41^. Normalizing or standardizing the features before clustering can aid in addressing this issue.

To overcome these limitations and enhance clustering performance on diverse datasets, researchers have developed extensions and variations of *k*-means clustering, such as *k*-medoids (PAM)^42,43^, fuzzy *k*-means^44^, and hierarchical *k*-means^45^.

### Research gaps

Prior research on MCDM has established the groundwork for addressing practical decision-making challenges across various fields. Although significant progress has been made, notable gaps remain in existing research.

First, decision-makers often encounter difficulties in achieving efficient and stable final decisions with reduced uncertainty when model options are limited, particularly when alternative samples are scarce. Second, most existing MCDM methodologies emphasize the aggregation phase while overlooking the crucial steps of grouping, deconstruction, and decomposition. These steps are vital for augmenting decision support, especially in specialized contexts. Third, several solutions designed to address the clustering uncertainty in *k*-means clustering are tailored for large-sample scenarios, leaving a void in solutions appropriate for small-sample situations. This issue is compounded by the ambiguous classification of marginal individuals in small samples, which complicates the decision-making process. Additionally, despite global concerns about transport safety, the OAS lacks a comprehensive assessment system, relying instead on fragmented research. Although this approach may be beneficial for individual countries, it impedes a holistic analysis of transport safety strengths and weaknesses across the OAS, each with its distinct contexts and conditions.

To address these gaps, this study introduces a hybrid MCDM methodology that integrates a machine learning tool (i.e., a graph-based technique). This model incorporates multiple stages of MCDM—weighting, aggregating, and grouping—along with deconstruction and decomposition into a streamlined process. This approach aims to generate consistently stable, reliable, and effective decision-making outcomes at a broader regional level. It addresses the complexities of MCDM that arise from intertwined sensitivity and uncertainty, thereby offering a more comprehensive solution.

## Methodology

### DCRITIC

DCRITIC is a modified version of the CRITIC method that was proposed by^46^. This adaptation, named distance correlation-based CRITIC, combines distance correlation with the established CRITIC method^47^. The method assesses the significance of the criteria by considering the distance correlations among them.

DCRITIC offers a structured methodology for exploring the relationships among criteria and prioritizing them based on their impact on decision outcomes. It proves especially valuable in complex decision-making scenarios where interactions and dependencies among criteria are pivotal^48^.

### WASPAS

The WASPAS method is proposed by^49,50^ and is employed in MCDM to evaluate alternatives based on multiple criteria. This method involves assigning weights to various metrics or criteria to reflect their relative importance in decision-making processes. A comprehensive score is then calculated by multiplying the score of each indicator by its corresponding weight. Essentially, WASPAS integrates the advantages of both Weighted Aggregated Sum (WAS) and Weighted Product Model (WPM), thereby providing flexibility in method selection depending on the context and offering a more holistic approach.

The WASPAS method offers a structured approach to decision-making by integrating weighted criteria into a comprehensive assessment of alternatives. It is widely applied in fields such as engineering, management, and finance to facilitate decision-making processes that require simultaneous consideration of multiple criteria.

### Improvement of *k*-means with machine learning

The conventional *k*-means clustering method often encounters challenges in selecting initial cluster centers, which are crucial for its overall performance. Despite the availability of numerous heuristic methods to determine these centers, none consistently yield an optimal solution.

In response to this challenge, we propose a novel approach that combines a machine learning tool (i.e., graph-based technique) with the *k*-means algorithm, addressing the limitations of current methods and those inherent in *k*-means itself. Although the graph method was not initially intended for determining initial centers, its application in this context has proven beneficial, especially for complex datasets, by enhancing robustness and effectively capturing the inherent cluster structure.

The present approach employs a graph-based technique to analyze the data prior to initializing the *k*-means algorithm. This preprocessing step enables the identification of more representative initial centers based on actual data relationships, rather than relying on random or heuristic selections. Unlike traditional initialization strategies (e.g., random seeding, k-means++, or heuristic-based methods), our approach leverages spectral properties of a similarity graph constructed from the decision matrix. By computing the Laplacian matrix and extracting its eigenvectors, we project the data into a lower-dimensional space that better preserves intrinsic structure. This enables more representative and stable centroid initialization, particularly for datasets with complex, non-spherical, or noisy patterns—limitations often faced by conventional methods. This method is especially advantageous for complex datasets where inherent cluster structures are not readily apparent, thus increasing the robustness and accuracy of the *k*-means clustering.

By integrating these techniques, the proposed method not only addresses the fundamental issues associated with the selection of initial centers in *k*-means clustering but also improves the overall clustering process.

### DCRITIC–WASPAS–K-means model with a graph-based technique

The proposed DCRITIC–WASPAS–K-means model integrates three key components into a unified MCDM framework. First, the DCRITIC method objectively determines the weights of criteria by analyzing the variability and interdependence among indicators. Second, the WASPAS method aggregates weighted criteria to generate performance scores for each alternative. Third, a graph-based technique is introduced to enhance the traditional k-means clustering by selecting initial centroids through spectral analysis of the data’s similarity structure.

**Step 1.** For the dataset, the decision matrix is created.

1 $$\begin{array}{*{20}{c}} {X=\left[ {\begin{array}{*{20}{c}} {{x_{11}}}& \cdots &{{x_{i1}}} \\ \vdots & \ddots & \vdots \\ {{x_{1j}}}& \cdots &{{x_{ij}}} \end{array}} \right]~} \end{array}$$

where ${x_{ij}}$ denotes the value of the *i* scheme under the *j* indicator.

**Step 2.** The logical functions are used to standardize the data.

2 $$\begin{array}{*{20}{c}} {{x_{ij}}=\frac{1}{{1+{e^{{x_{ij}}}}}}~} \end{array}$$

where *x* denotes the value of our dataset.

**Step 3.** The standard deviation of each criterion is calculated.

3 $$\begin{array}{*{20}{c}} {{t_j}=\sqrt {\frac{{{{\left( {\mathop \sum \nolimits_{{i=1}}^{m} {x_{ij}} - \overline {{{x_j}}} } \right)}^2}}}{{m - 1}}} ~} \end{array}$$

where *t* denotes the standard deviation, $\overline {{{x_j}}}$ indicates the mean score of criteria *j*, and *m* represents the total number of alternatives.

**Step 4.** The distance correlation of every pair of criteria is calculated.

4 $$\begin{array}{*{20}{c}} {dCor\left( {{o_j},{o_{{j^\prime }}}} \right)=\frac{{dCov\left( {{o_j},{o_{{j^\prime }}}} \right)}}{{sqrt{{\left( {dVar\left( {{o_j}} \right)dVar\left( {{o_{j^{\prime}}}} \right)} \right)}^\prime }}}~} \end{array}$$

where $dCov\left( {{o_j},{o_{j^{\prime}}}} \right)$ denotes the distance covariance between ${o_j}$ and ${o_{j'}}$, $dVar\left( {{o_j}} \right)$=$dCov\left( {{o_j},{o_j}} \right)$ represents the distance variance of ${o_j}$, and $dVar\left( {{o_{j'}}} \right)$=$dCov\left( {{o_{j'}},{o_{j'}}} \right)$ denotes the distance variance of ${o_{j'}}$.

**Step 5.** The information content is computed.

5 $$\begin{array}{*{20}{c}} {{I_j}={T_j}\mathop \sum \limits_{{j^{\prime}=1}}^{n} \left( {1 - dCor\left( {{o_j},{o_{j^{\prime}}}} \right)} \right)} \end{array}$$

where ${I_j}$ denotes the information content of ${o_j}$.

**Step 6.** The objective weights of the SPIs are determined.

6 $$\begin{array}{*{20}{c}} {{w_j}=\frac{{{I_j}}}{{\mathop \sum \nolimits_{{j=1}}^{m} {I_j}^{\prime }}}~} \end{array}$$

where ${{\text{w}}_{\text{j}}}$ denotes the objective weight of ${{\text{o}}_{\text{j}}}$.

**Step 7.** For ${x_{ij}}$ and ${w_j}$, was used to calculate the weighted sum and product, respectively.

7 $$\begin{array}{*{20}{c}} {WS=\mathop \sum \limits_{{j=1}}^{n} {w_j}*{x_{ij}}~} \end{array}$$

8 $$\begin{array}{*{20}{c}} {WP=\mathop \prod \limits_{{j=1}}^{n} {{\left( {{x_{ij}}} \right)}^{{w_j}}}~} \end{array}$$

where WS and WP denote the weighted sum and weighted product, respectively.

**Step 8.** The scores are consolidated.

9 $$\begin{array}{*{20}{c}} {W{Q_i}=\lambda \times W{S_i}+\left( {1 - \lambda } \right) \times W{P_i}~} \end{array}$$

where WQ denotes the final combination score, λ represents a parameter between 0 and 1 that balances the weighted sum and the weighted product.

**Step 9.** The similarity matrix S is constructed as follows:

10 $$\begin{array}{*{20}{c}} {s\left( {{x_i},{x_j}} \right)=\exp \left( { - \frac{{{{\left\| {{x_i} - {x_j}} \right\|}^2}}}{{2{\sigma ^2}}}} \right)} \end{array}$$

The element ${S_{ij}}$ of the similar matrix S of the entire data set is $s\left( {{x_i},{x_j}} \right)$.

**Step 10.** The Laplacian matrix L is constructed:

11 $$\begin{array}{*{20}{c}} {{D_{II}}=\mathop \sum \limits_{J} {S_{ij}}} \end{array}$$

12 $$\begin{array}{*{20}{c}} {L=D - S} \end{array}$$

**Step 11.** The eigenvector of the Laplacian matrix is computed.

The eigenvector corresponding to the first *k* minimum eigenvalues of matrix *L* is computed, and matrix *U* is formed.

13 $$\begin{array}{*{20}{c}} {U=\left\{ {{u_1},{u_2}, \ldots \ldots {u_k}} \right\}} \end{array}$$

**Step 12.** The center is located.

The representation of the original data points was converted into a representation in the eigenvector space. Using the converted data and the *k*-means method, *k* initial centers were located.

14 $$\begin{array}{*{20}{c}} {{C_{init}}=\left\{ {{c_1},{c_2}, \ldots {c_k}} \right\}} \end{array}$$

**Step 13.** Using the centers obtained in **Step 12**, the final grouping is derived.

For each data point ${x_i}$, its distance from each center ${c_j}$ was calculated, and it was then assigned to the nearest center.

15 $$\begin{array}{*{20}{c}} {l\left( i \right)=argmi{n_j}{{\left\| {{x_i} - {c_j}} \right\|}^2}} \end{array}$$

For each central ${c_j}$, its position is updated using the mean of all assigned points.

16 $$\begin{array}{*{20}{c}} {{c_j}=\frac{1}{{\left| {{S_j}} \right|}}\mathop \sum \limits_{{{x_i} \in {S_j}}} {x_i}} \end{array}$$

Equations (15) and (16) are repeated until the algorithm converges, at which point each data point is assigned to a center, forming the final grouping, where ${S_j}$ denotes the set of all data points assigned to the central ${c_j}$.

## Results and discussion

### Case study

#### Safety performance indicators (SPIs)

SPIs are essential in various domains, including transport safety engineering. They enable researchers and organizations to evaluate safety performance, providing insights into the frequency, trends, and patterns of security incidents. SPIs set benchmarks that form the basis for evaluation and help organizations identify achievable safety goals. Additionally, they play a crucial role in risk identification and the mitigation of potential security hazards.

**Fig. 1:**
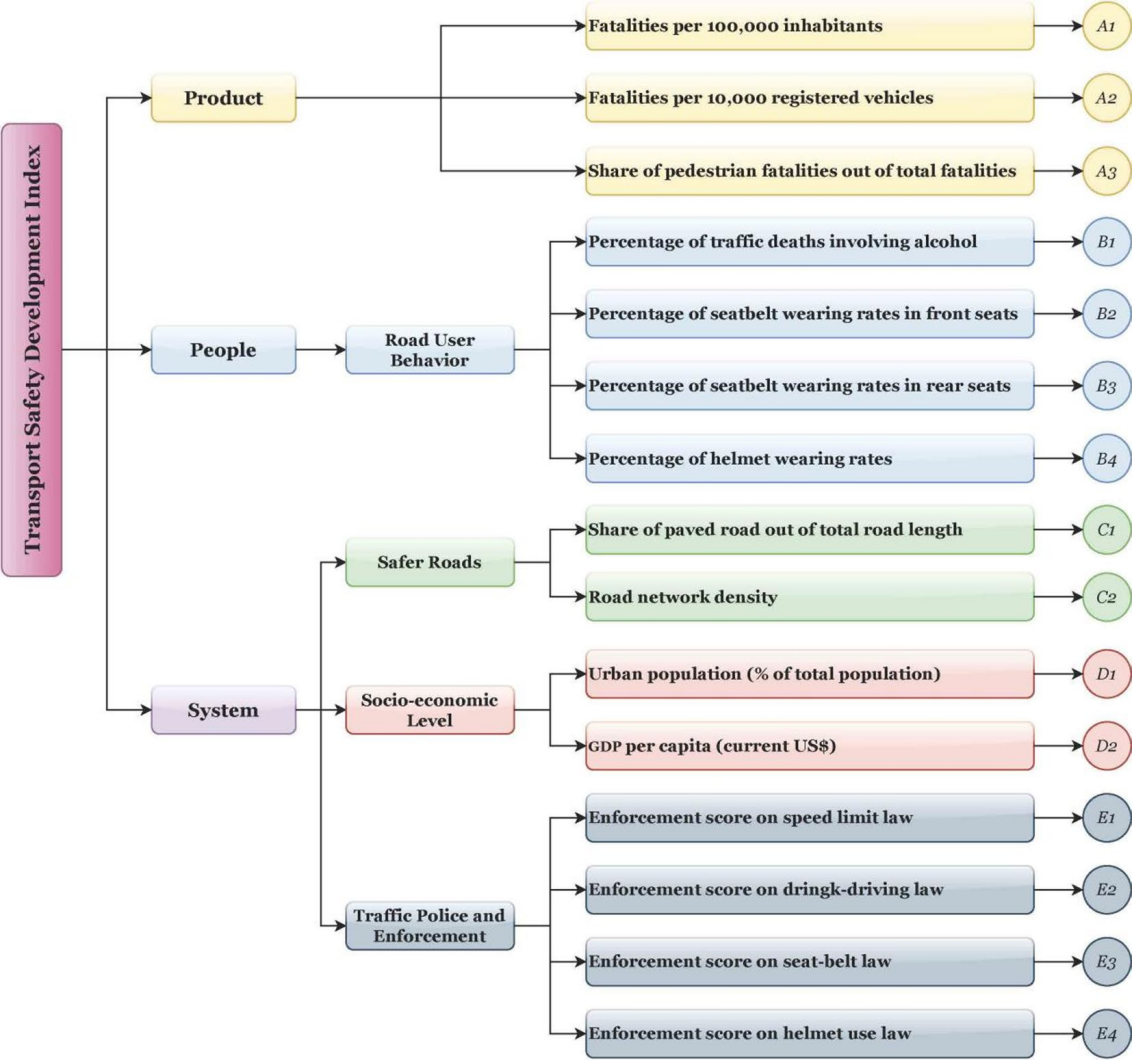
SPIs constructed by this study.

In this study, the SPIs framework comprises 15 indicators derived from the human–vehicle–road–environment–management system^51–55^. The SPIs framework is illustrated in Fig. 1.

#### Data sources

Data for SPIs from the years 2010 and 2020 were collected from multiple sources, including authoritative reports and official databases for the OAS member states, including AG (Antigua and Barbuda), AR (Argentina), BB (Barbados), BO (Bolivia), BR (Brazil), BS (The Bahamas), BZ (Belize), CA (Canada), CL (Chile), CO (Colombia), CR (Costa Rica), CU (Cuba), DM (Dominica), DO (Dominican Republic), EC (Ecuador), GD (Grenada), GT (Guatemala), GY (Guyana), HN (Honduras), HT (Haiti), JM (Jamaica), KN (St. Kitts and Nevis), LC (Saint Lucia), MX (Mexico), NI (Nicaragua), PA (Panama), PE (Peru), PY (Paraguay), SR (Suriname), SV (El Salvador), TT (Trinidad and Tobago), US (United States), UY (Uruguay), VC (Saint Vincent and the Grenadines), and VE (Venezuela).

Specifically, indicators A1, B1, and B2 were sourced from the WHO Global Health Observatory^56^, whereas A2 was calculated based on WHO data concerning road fatalities and registered motor vehicles^56^. C1 was obtained from the library of the Central Intelligence Agency^57^ and ChartsBin^58^, while C2 was calculated using the total length of road network and area of land data from the World Bank^59,60^, ChartsBin^58^, and Statista^61^. the World Bank database^59,60^ served as the source for extracting indicators D1 and D2, and the Global Status Report on Road Safety^62–65^ provided data for indicators B3, B4, E1, E2, E3, and E4. The human development index (HDI) was derived from the Annual Human Development Report of the United Nations Development Program^66,67^.

### Computational results

#### Ranking

By employing the proposed DCRITIC–WASPAS–K-means model, transport safety scores of the OAS countries and their corresponding rankings are presented in Table 1.

**Table 1:** Rankings of OAS countries.

Country	2010		2020	
	Score	Rank	Score	Rank
AG	0.5085	7	0.4290	11
AR	0.2041	30	0.3830	15
BB	0.5572	4	0.5267	6
BZ	0.2559	25	0.3869	13
BO	0.1940	32	0.1772	35
BR	0.2929	19	0.5192	8
CA	0.5939	3	0.5557	2
CL	0.3262	16	0.4703	9
CO	0.4405	12	0.3038	20
CR	0.5543	5	0.2430	28
CU	0.5445	6	0.2623	27
DM	0.4163	13	0.2019	30
DO	0.3013	18	0.2011	32
EC	0.2724	22	0.395	12
SV	0.2373	27	0.2686	25
GD	0.4717	11	0.2149	29
GT	0.2768	21	0.2872	23
GY	0.1893	34	0.2955	21
HT	0.1766	35	0.1852	33
HN	0.3076	17	0.339	19
JM	0.2092	29	0.2649	26
MX	0.1954	31	0.3766	16
NI	0.2682	23	0.3849	14
PA	0.4835	9	0.3473	18
PY	0.3378	15	0.2927	22
PE	0.2204	28	0.1838	34
LC	0.2391	26	0.2013	31
VC	0.7107	1	0.5496	3
KN	0.4864	8	0.4490	10
SR	0.4135	14	0.276	24
BS	0.2631	24	0.5287	5
TT	0.4807	10	0.5351	4
US	0.6219	2	0.6161	1
UY	0.1917	33	0.5235	7
VE	0.2778	20	0.3569	17

As listed in Table 1, US, CA, and VC have consistently excelled and ranked highly in terms of transport safety over the past ten years, reflecting their commendable achievements. In contrast, BO and HT consistently ranked lower, highlighting substantial challenges and opportunities for enhancement in their transport safety efforts. UY notably improved its rankings over two years, demonstrating substantial progress in improving transport safety outcomes, whereas the sharp decline in the ranking of CR signifies the importance of focusing more on transport safety measures.

#### Grouping

Developing effective action plans and setting goals requires learning from and benchmarking against high-performing peers. However, owing to varying contexts among countries, directly emulating the top performers may not be feasible for those who are less advanced. Therefore, it is crucial to categorize countries with similarity and assess their strengths and weaknesses within comparable groups. Using the proposed k-means with a graph-based technique based on the safety score (DCRITIC–WASPAS score), the countries are classified into six groups. The corresponding groupings of OAS countries are listed in Table 2.

**Table 2:** Groups of the OAS countries.

Country	2010	2020
	Group	Group
AG	2	1
AR	0	5
BB	2	1
BZ	0	5
BO	4	2
BR	5	5
CA	1	4
CL	1	0
CO	5	0
CR	2	3
CU	2	0
DM	2	5
DO	5	5
EC	4	0
SV	2	5
GD	3	1
GT	1	0
GY	1	2
HT	4	5
HN	1	5
JM	3	1
MX	5	5
NI	4	0
PA	5	2
PY	1	2
PE	0	2
LC	5	3
VC	3	1
KN	3	1
SR	1	2
BS	4	4
TT	2	1
US	2	4
UY	0	0
VE	5	5

Table 2 indicates that several countries have retained the same classification over the past decade. However, certain countries, such as AR and BZ, have moved from the first class (highest performance) to the last class (lowest performance). Notably, NI and PA have achieved significant progress in enhancing their transport safety performance.

### Robust test

#### Comparison of ranking

1. Initial sensitivity.

To examine the initial sensitivity of the proposed model, we contrast the rankings derived from different normalization methods, i.e., Fuzzy Quantization (FQ), Decimal Scaling (DS), and Vector (VE), as displayed in Table 3.

**Table 3:** Contrasts of rankings across different ***normalization*** methods.

Country	2010			2020		
	Fuzzy quantization	Decimal scaling	Vector	Fuzzy quantization	Decimal scaling	Vector
AG	7	12	5	11	16	6
AR	30	33	28	15	13	13
BB	4	6	2	6	9	2
BZ	25	22	19	13	12	12
BO	32	32	33	35	35	33
BR	19	23	27	8	5	10
CA	3	2	4	2	1	3
CL	16	16	16	9	8	11
CO	12	8	13	20	20	22
CR	5	4	9	28	27	26
CU	6	5	10	27	25	29
DM	13	14	11	30	32	27
DO	18	17	15	32	31	34
EC	22	19	21	12	15	18
SV	27	25	29	25	28	31
GD	11	13	6	29	30	25
GT	21	20	18	23	23	21
GY	34	29	35	21	19	23
HT	35	34	34	33	29	35
HN	17	18	20	19	21	20
JM	29	35	25	26	26	16
MX	31	31	31	16	14	14
NI	23	21	22	14	11	17
PA	9	7	12	18	18	15
PY	15	15	17	22	22	24
PE	28	30	32	34	33	32
LC	26	26	24	31	34	30
VC	1	1	1	3	7	5
KN	8	11	7	10	10	8
SR	14	10	14	24	24	28
BS	24	28	23	5	3	7
TT	10	9	8	4	4	4
US	2	3	3	1	2	1
UY	33	27	30	7	6	9
VE	20	23	26	17	17	19

**Fig. 2:**
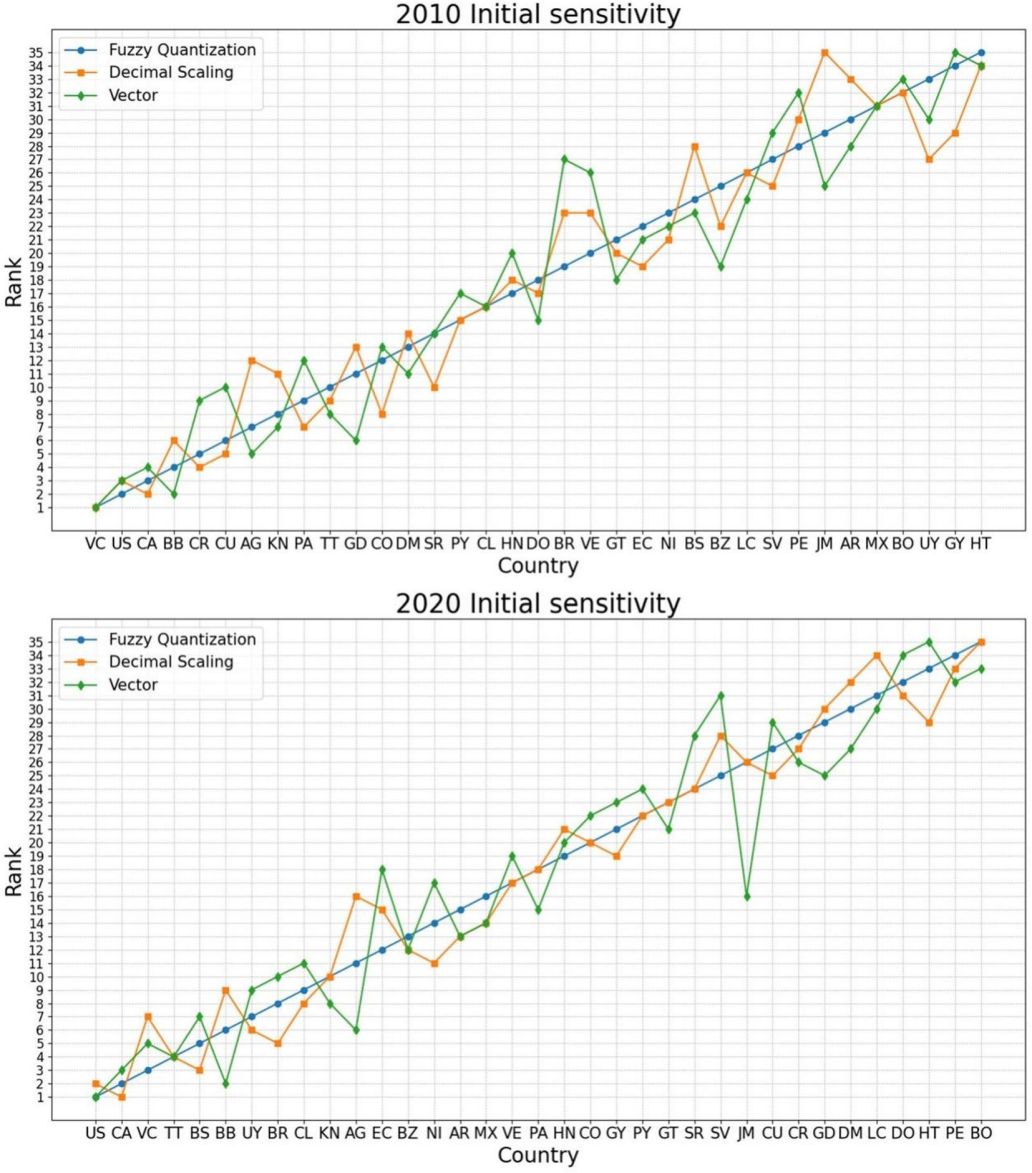
Ranking contrasts across different ***normalization*** methods (years 2010 and 2020).

Table 3; Fig. 2 demonstrate that the OAS countries’ rankings generated through the DCRITIC–WASPAS–K-means model are fairly consistent across the various normalization approaches. The closely intertwined ranking lines in Fig. 2 support this observation.

**Fig. 3:**
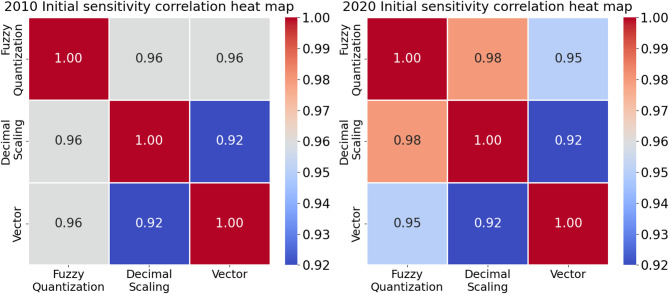
Heat map of correlation between the rankings based on different ***nomalization*** methods.

To objectively and concretely quantify the similarity of the rankings derived from different normalization methods, we performed correlation analyses, as depicted in Fig. 3.

As depicted in Fig. 3, the Spearman’s correlation coefficients between the rankings using the three normalization methods consistently exceeded 0.85, indicating a strong consensus and demonstrating the robustness of the proposed model.

2. Internal creditability.

To examine the internal credibility of the proposed model, we contrast the rankings derived from different weighting methods, specifically DCRITIC, Entropy^68^, and MEthod based on the Removal Effects of Criteria (MEREC)^69^, as presented in Table 4.

**Table 4:** Contrasts of rankings across different ***weighting*** methods.

Country	2010			2020		
	DCRITIC	Entropy	MEREC	DCRITIC	Entropy	MEREC
AG	5	1	2	1	8	1
AR	31	21	25	22	16	20
BB	6	3	4	2	12	2
BZ	33	22	34	23	25	27
BO	32	35	33	33	32	34
BR	17	12	19	13	18	5
CA	13	10	10	12	7	3
CL	23	20	28	19	23	22
CO	21	18	24	30	5	26
CR	10	11	12	32	19	14
CU	12	15	14	11	10	13
DM	8	7	11	14	24	11
DO	16	13	16	15	26	17
EC	27	31	18	25	28	28
SV	11	23	13	20	29	21
GD	3	6	6	6	1	8
GT	25	19	30	24	21	32
GY	22	34	27	27	27	25
HT	30	33	32	31	34	33
HN	20	25	17	21	22	31
JM	2	9	5	8	20	12
MX	24	16	26	16	13	18
NI	28	26	31	17	17	24
PA	15	17	20	18	11	23
PY	26	27	29	29	15	29
PE	34	32	35	35	33	35
LC	14	28	8	34	35	16
VC	4	4	3	3	9	9
KN	1	2	1	4	4	10
SR	19	29	21	26	30	19
BS	35	30	22	10	6	7
TT	7	8	9	5	3	6
US	9	5	7	7	2	4
UY	29	14	15	9	14	15
VE	18	24	23	28	31	30

**Fig. 4:**
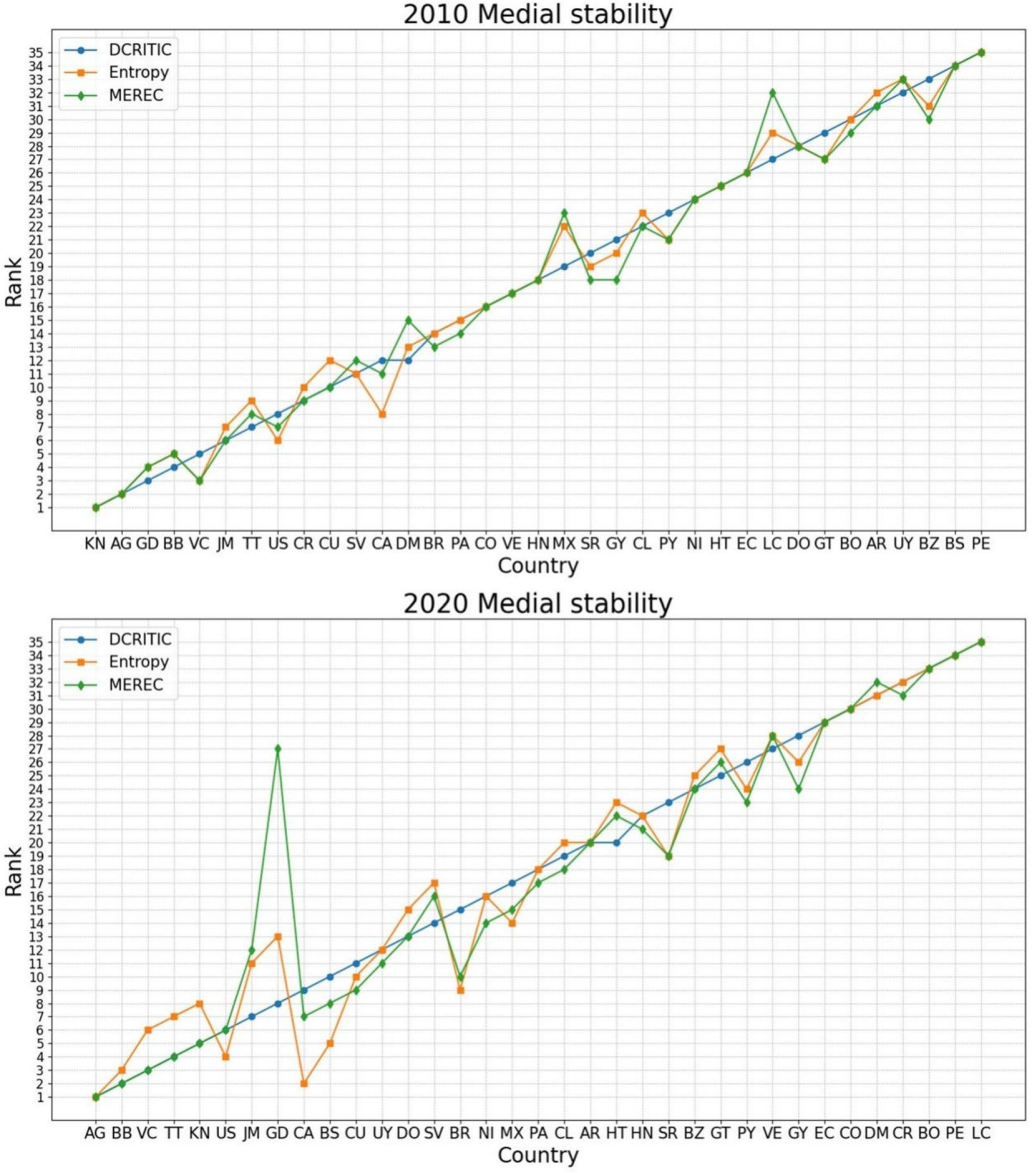
Ranking contrasts across different ***weighting*** techniques (years 2010 and 2020).

As illustrated in Table 4 and the ranking line chart in Fig. 4, the rankings of OAS countries using the DCRITIC–WASPAS–K-means model show considerable consistency across the various weighting methods. The closely intertwined ranking lines in Fig. 4 demonstrate this finding.

**Fig. 5:**
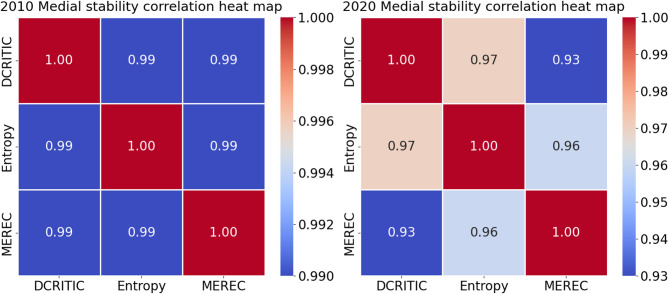
Heat map of correlation between the rankings based on different ***weighting*** methods.

To more objectively and concretely quantify the similarity of these rankings, correlation analyses were performed, as displayed in Fig. 5.

As observed in Fig. 5, the rankings obtained from the three different weighting techniques exhibited Spearman’s correlation coefficients greater than 0.8. This high correlation suggests a strong agreement between the methods, and thus, supports the validity of the proposed model.

3. Lateral reliability.

To evaluate the external reliability of the proposed model, we contrast the rankings generated by the WASPAS method with those produced by other classical aggregation approaches, i.e., TOPSIS^70^ and VIKOR^71^, as listed in Table 5.

**Table 5:** Contrasts of rankings across different ***aggregating*** methods.

Country	2010			2020		
	WASPAS	TOPSIS	VIKOR	WASPAS	TOPSIS	VIKOR
AG	5	4	3	1	1	2
AR	31	31	30	22	21	17
BB	6	3	1	2	2	1
BZ	33	34	34	23	27	25
BO	32	32	31	33	34	33
BR	17	17	16	13	19	18
CA	13	14	13	12	15	11
CL	23	22	18	19	23	21
CO	21	19	20	30	33	32
CR	10	11	11	32	17	35
CU	12	12	12	11	10	10
DM	8	8	9	14	8	8
DO	16	15	22	15	12	14
EC	27	29	27	25	32	29
SV	11	13	14	20	14	16
GD	3	5	2	6	5	3
GT	25	27	26	24	31	27
GY	22	21	21	27	26	23
HT	30	28	29	31	30	31
HN	20	24	24	21	28	26
JM	2	2	6	8	6	5
MX	24	23	23	16	20	20
NI	28	26	28	17	18	24
PA	15	16	15	18	25	28
PY	26	25	25	29	29	30
PE	34	33	33	35	35	34
LC	14	9	8	34	16	15
VC	4	6	4	3	3	4
KN	1	1	5	4	7	7
SR	19	18	17	26	22	19
BS	35	35	35	10	13	12
TT	7	7	7	5	4	6
US	9	10	10	7	9	9
UY	29	30	32	9	11	13
VE	18	20	19	28	24	22

**Fig. 6:**
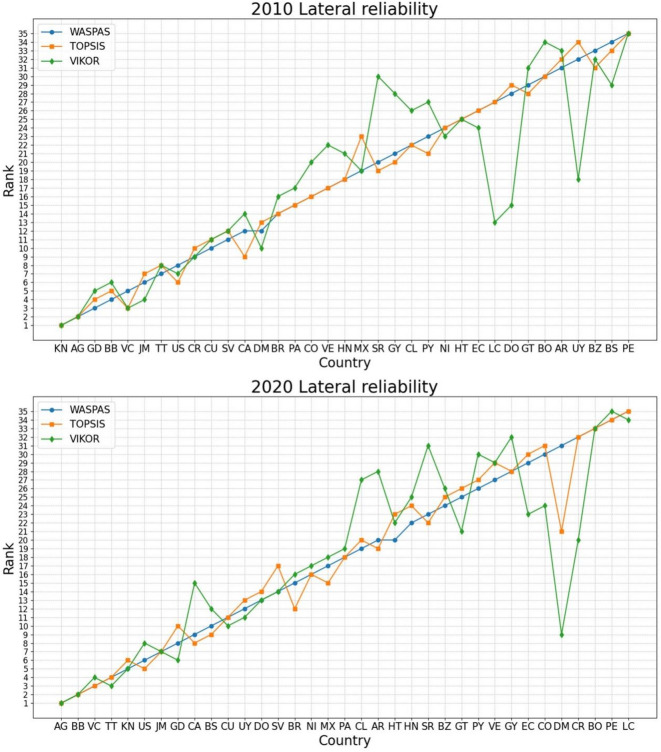
Ranking contrasts across different ***aggregating*** methods in the years 2010 and 2020.

As outlined in Table 5, the OAS countries’ rankings by means of the DCRITIC–WASPAS–K-means model exhibit a strong consistency across these aggregation approaches. Figure 6 uses line charts to visually represent the differences in rankings between the various aggregation methods, illustrating a convergence in the rankings through the interwoven lines.

**Fig. 7:**
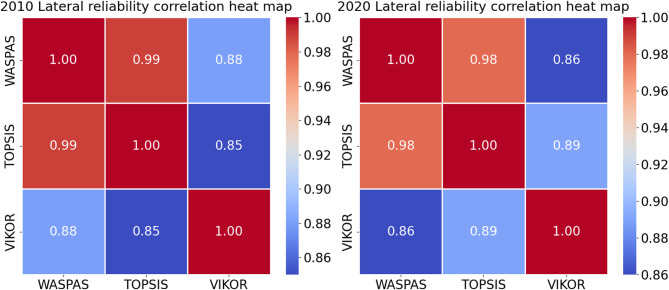
Heat map of correlation between the rankings based on different ***aggregating*** methods.

For a more objective and concrete quantification of the similarity in rankings across different aggregation methods, we conducted a correlation analysis, and the results are presented in Fig. 7.

As depicted in Fig. 7, the Spearman’s correlation coefficients among the various approaches consistently surpassed 0.8, indicating a strong correlation between WASPAS and the other two classical methods, which further suggests the reliability of the proposed model.

#### Comparison of grouping

1. Initial sensitivity.

To examine the internal stability of the proposed model, we comparatively analyzed the groupings derived from different normalization methods, as detailed in Table 6.

**Table 6:**
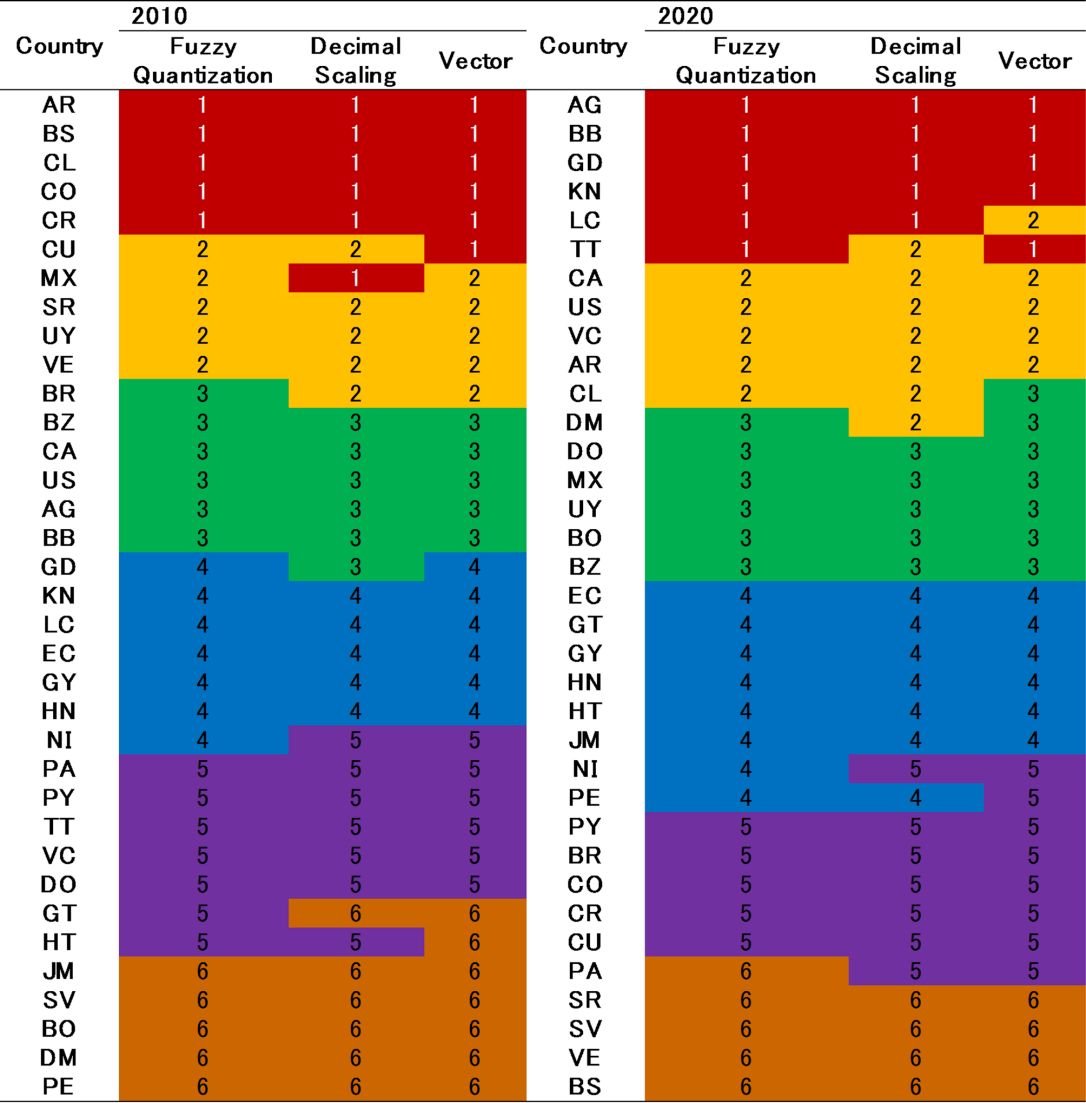
Contrasts of groups across different ***normalization*** methods.

As listed in Table 6, the country groupings were highly similar in 2010. Despite some variations in the 2020 results, a significant portion of countries remained consistently categorized across all three methods.

To further measure groups similarities across various normalization techniques, the V-measure analyses were conducted, as shown in Table 7.

**Table 7:** Grouping similarities across different normalization techniques.

V-measure	2020			2010		
	Fuzzy quantization	Decimal scaling	Vector	Fuzzy quantization	Decimal scaling	Vector
Fuzzy quantization	1			1		
Decimal scaling	0.812	1		0.831	1	
Vector	0.809	0.756	1	0.821	0.737	1

As shown in Table 7, the coefficients of V-measure between groups under the three normalization techniques are considerably high in the two years, reflecting strong similarities. This demonstrates the stability of *k*-means clustering with a graph-based technique as a grouping method.

2. Lateral reliability.

To examine the external reliability of the proposed model, we contrast the groupings from *k*-means using a graph-based technique, Density-Based Spatial Clustering of Applications with Noise (DBSCAN)^72^, and Gaussian mixture models (GMM)^73^, as presented in Table 8.

**Table 8:**
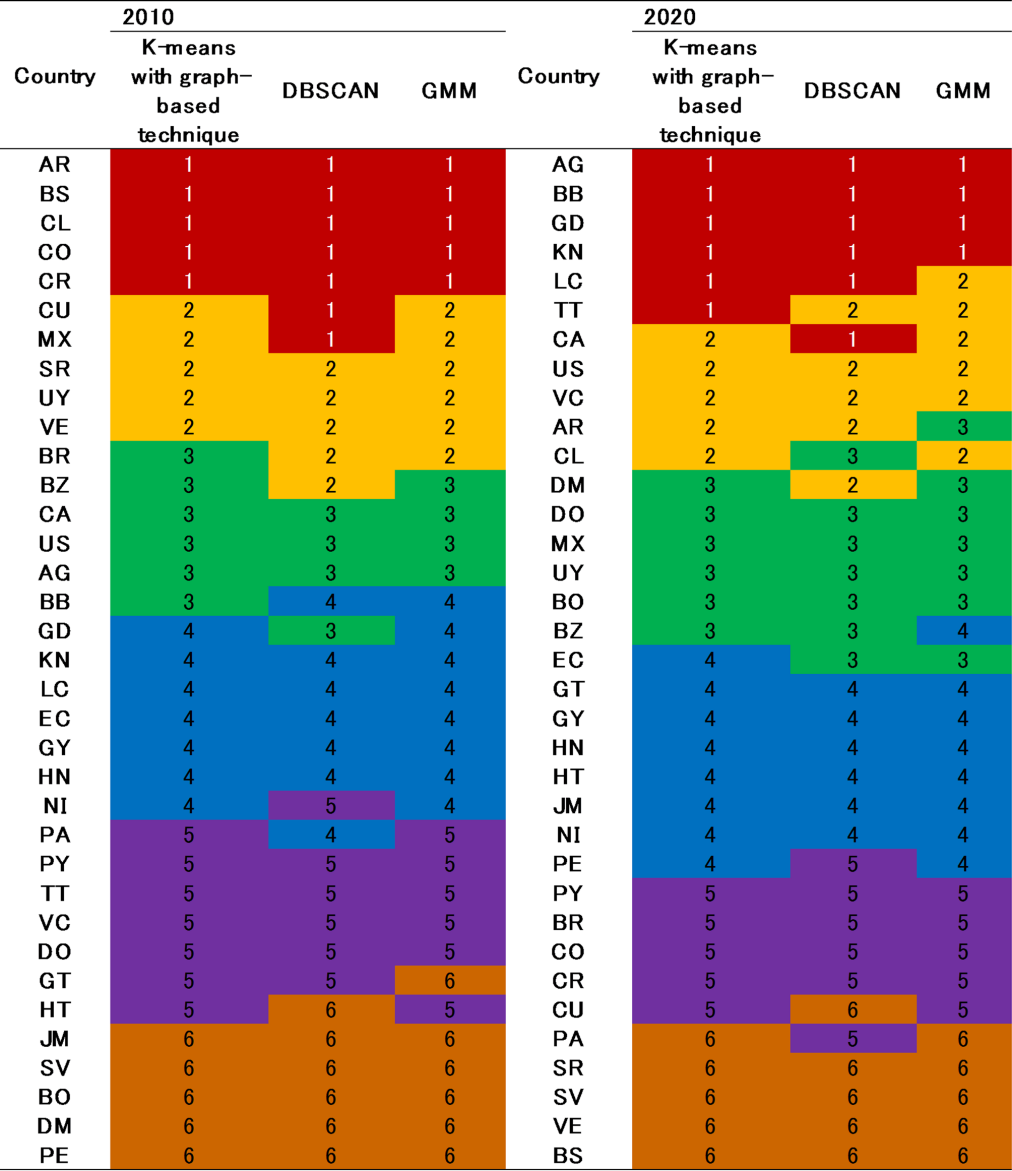
Contrasts of groups across different ***grouping*** approaches.

As indicated in Table 8, in 2010, the country groupings were largely consistent with minimal variations. Although some discrepancies were observed in the 2020 results, a considerable proportion of countries remained consistently classified across all three methods.

To further measure the similarities between groups by the proposed model and other benchmarking grouping techniques, the V-measure analyses were conducted, as shown in Table 9.

**Table 9:** Grouping similarities across different normalization techniques.

V-measure	2020			2010		
	K-means with graph- based technique	DBSCAN	GMM	K-means with graph- based technique	DBSCAN	GMM
K-means with graph- based technique	1			1		
DBSCAN	0.728	1		0.789	1	
GMM	0.707	0.711	1	0.724	0.775	1

As shown in Table 9, the V-measure coefficients between groups by the proposed model and the other two benchmarking models are quite high in the two years. This confirms the reliability and practicality of *k*-means with graph-based techniques as a robust method.

## Practical implications

### Dynamic evolution of SPIs

**Fig. 8:**
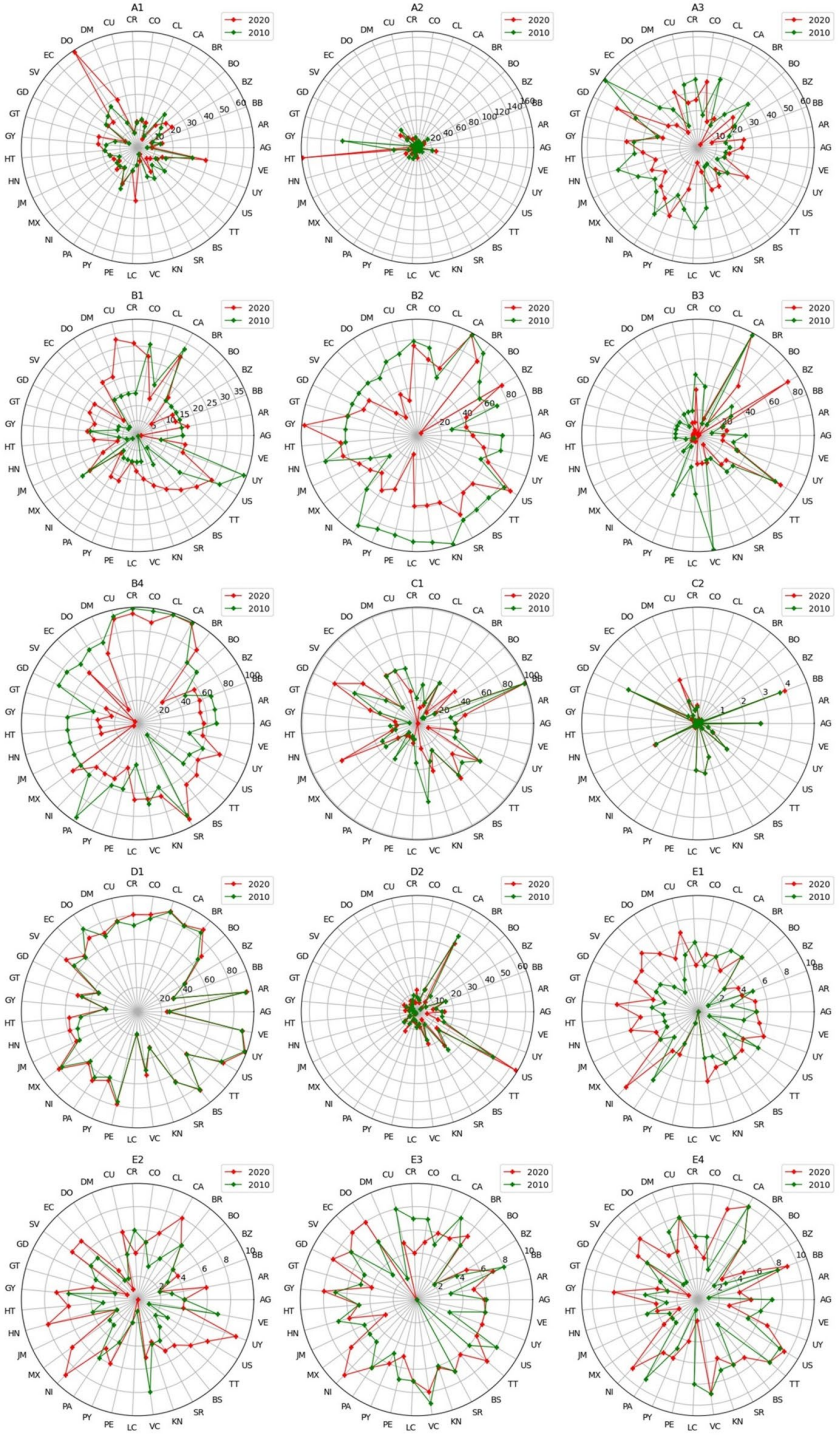
Dynamic evolution of the SPIs over the past ten years.

To analyze the evolution of specific SPIs over time, we created radar plots, as depicted in Fig. 8. In the radar plots, each axis corresponds to a different country, with the distance from the center reflecting that country’s performance score changes on a specific criterion. Tracking these changes enables ongoing performance assessment and adaptation to evaluate conditions. For benchmarking, decision-making, or policy evaluation, recognizing dynamic shifts is essential to respond to trends, enhance performance, and adjust strategies effectively.

Figure 8 illustrates that most countries within the OAS consistently demonstrated improvements in transport safety performance across various indicators. This trend underscores the increasing focus on transport safety development among OAS member countries. For example, Guyana (GY) displayed significant improvement in indicator *A*_*2*_, whereas El Salvador (SV) and Saint Lucia (LC) exhibited notable improvements in indicator *A*_*3*_. Most countries exhibit continuous improvements in indicators *D*_*1*_, *E*_*1*_, and *E*_*2*_. For indicator *D*_2_, which measures GDP per capita, all nations except Canada (CA) and the United States (US) remained relatively unchanged over the past decade. However, certain countries experienced notable regressions in specific areas. The Dominican Republic (DO) showed a decline in indicator *A*_*1*_, and Haiti (HT) regressed on indicator *A*_*2*_. Additionally, for indicators *B*_*2*_ and *B*_*3*_, which measure the percentage of seatbelt usage in the front and rear seats, respectively, most states displayed a regressive trend. The most significant declines were observed in Peru (PE) for *B*_*2*_ and Saint Vincent and the Grenadines (VC) for *B*_*3*_.

### Deconstruction of composite score

**Fig. 9:**
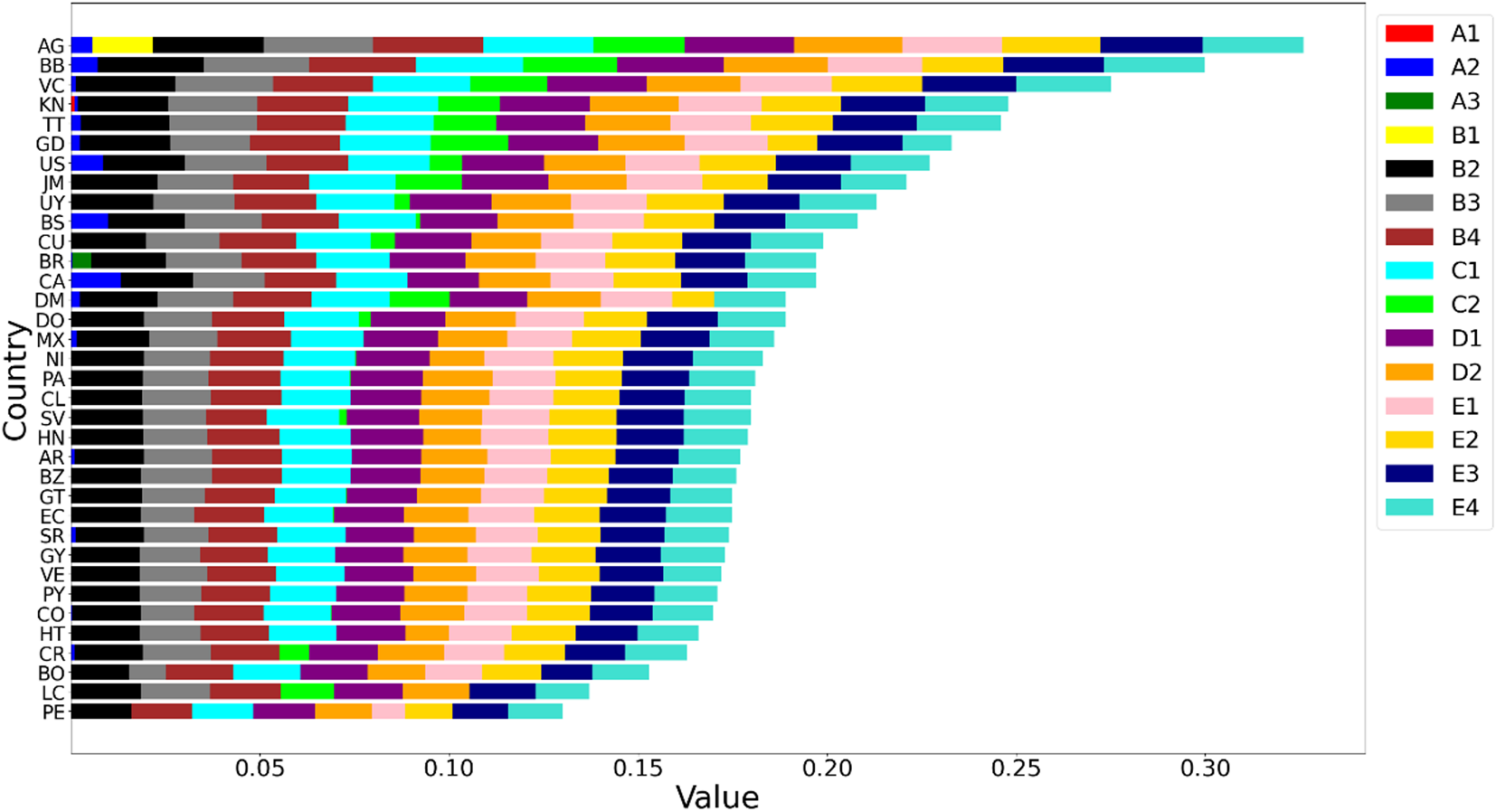
Breaking down the overall safety score to highlight the specific contributions of individual indicators.

To understand the impact of each SPI on the overall index, we divided the index into individual components, as depicted in Fig. 9. This division aids in analyzing the strengths and weaknesses of the index, identifying key drivers of the index score, and assessing the relevance of each component.

As depicted in Fig. 9, AG, BB, and VC achieved the highest overall safety scores. Several key indicators significantly contributed to these high scores. Notably, AG was uniquely influenced by indicator *B*_1_, which measures the percentage of traffic deaths involving alcohol. Other critical indicators included *A*_2_, assessing traffic fatalities; *B*_2_, *B*_3_, and *B*_4_, evaluating the percentage of seatbelt usage in front and rear seats and helmet usage, respectively; and *C*_2_, which gauges road network density. Additional influential indicators were *D*_2_, reflecting the socioeconomic levels of the nations and underpinning funding for transport safety strategies, and *E*_1_–*E*_4_, which assess the enforcement of various traffic laws, thereby enhancing the transport safety performance in these three countries.

In addition to the top three countries, several nations demonstrated commendable performance on specific indicators that did not significantly impact the leading performers. For instance, KN, ranked fourth, excelled in indicator *A*_1_. BR, ranked twelfth, was notable for *A*_3_. These findings highlight the necessity for enhanced transport safety strategies and their implementation, even in high-performing countries. This situation offers opportunities for shared learning and improvement across nations. Moreover, these indicators emphasize the urgent need for countries to strengthen transport safety measures, reduce fatality rates, and expand road network density to ensure safer roads. Overall, this detailed analysis provides invaluable insights for policymakers and stakeholders, illuminating the complex dynamics of the composite index and supporting targeted interventions and improvements in critical areas for enhancing transport safety.

### Decomposition of overall score change

**Fig. 10:**
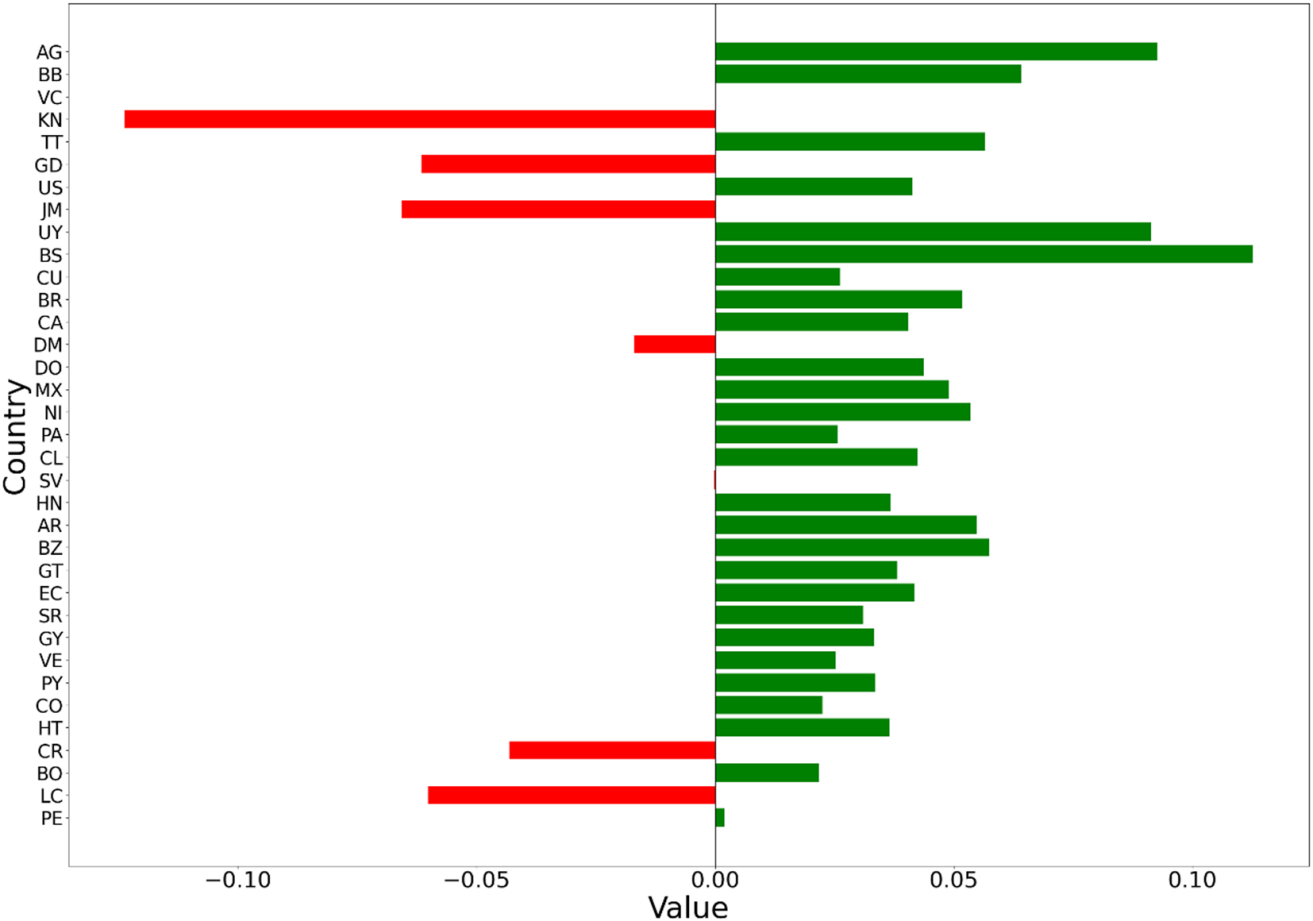
Transport safety achievement changes for each country from 2010 to 2020.

The overall change in achievement scores for each country from 2010 to 2020 is illustrated in Fig. 10.

As depicted in Fig. 10, AG, BS, and UY exhibited the most significant advancements in transportation safety, followed by BB, AR, and BZ. In contrast, PE and VC remained unchanged. DM and SV experienced slight regressions, whereas KN, JM, LC, and GD displayed the most notable regressions.

**Fig. 11:**
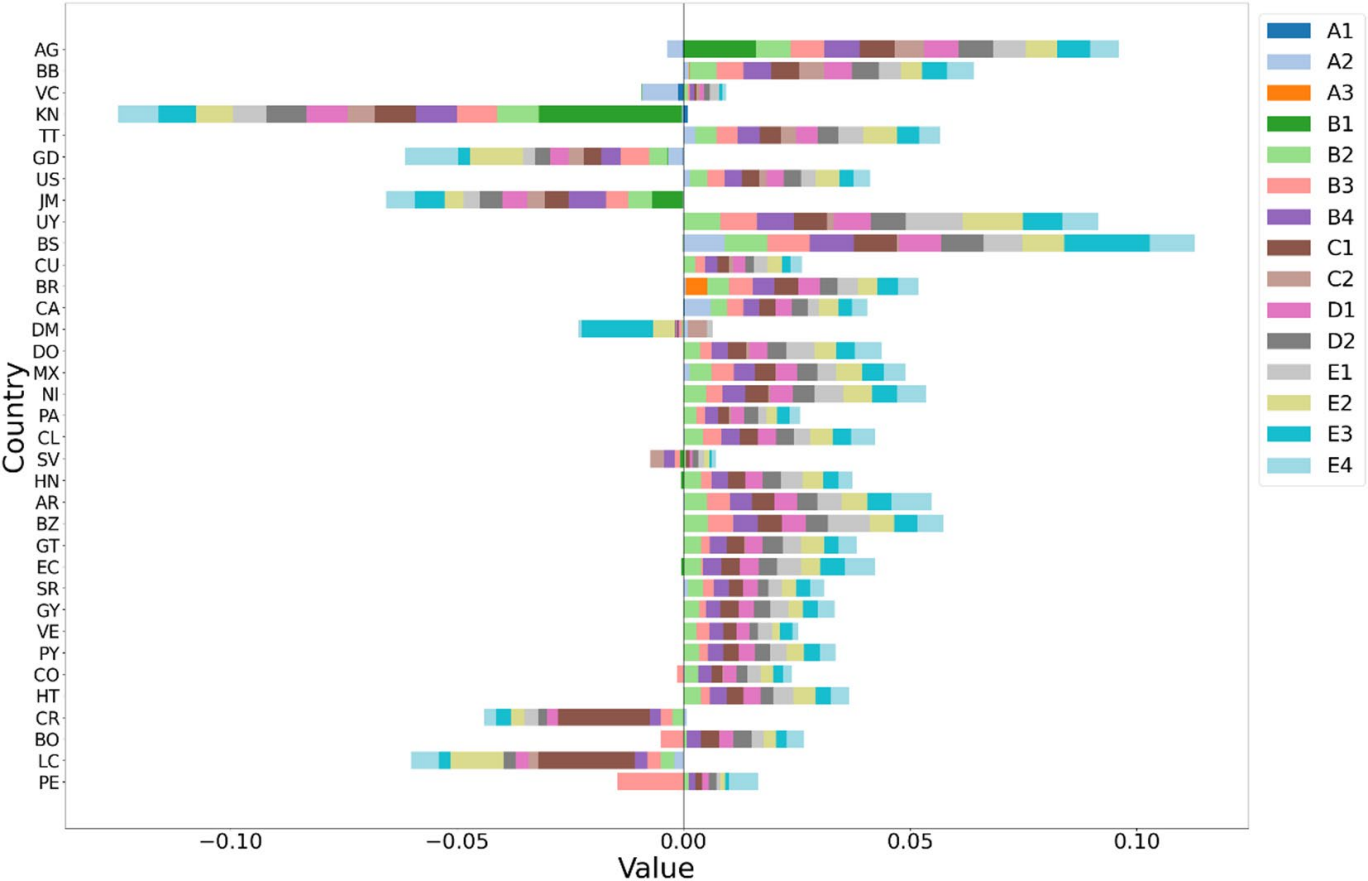
Contributions of each SPIs to the overall change in safety score.

To understand the drivers or sources of change in this holistic measure of achievement, we analyzed the factors or components that contributed to the changes in achievement between 2010 and 2020, as portrayed in Fig. 11. This analysis typically involves identifying and evaluating individual elements or sub-factors that influence the overall achievement and assessing their respective contributions to the observed changes.

Figure 11 illustrates that indicators *A*_1_, *A*_2_, and *A*_3_ are critical for evaluating fatalities and transportation safety, providing policymakers essential insights to evaluate the impacts of previous transportation policies. Within the specified timeframe, *A*_1_ showed no contribution to any nation except VC, where it had a negative impact. Indicator *A*_2_ increased in six countries, with BS exhibiting the most significant improvement. Additionally, *A*_3_ contributed positively to only BR and was a non-contributing factor in other countries.

Indicators *B*_1_, *B*_2_, *B*_3_, and *B*_4_ are essential for assessing road user behavior, which significantly influences transportation safety. *B*_1_, which measures alcohol consumption—a major factor in traffic-related fatalities—improved only in AG, while KN and JM experienced the most significant declines. The adoption of seatbelts in both front and rear seats, and the use of motorcycle helmets, measured by *B*_2_, *B*_3_, and *B*_4_ respectively, are crucial for individual protection. A positive trend in adherence to *B*_2_, *B*_3_, and *B*_4_ was observed across twenty-five countries, although KN, GD, and JM displayed noticeable declines in these metrics, with PE exhibiting considerable regression in *B*_3_.

Indicators *C*_1_ and *C*_2_, which are related to safer road infrastructure, are vital for vehicle operation and overall transportation safety. Twenty-eight nations reported improvements in *C*_1_, which measures the percentage of paved roads, with notable progress in BS, AG, UY, and BB. Conversely, CR and LC experienced significant declines in this indicator. Regarding road network density, represented by *C*_2_, 11 countries enhanced their road network density, with AG, BB, and DM showing the most remarkable advancements, whereas KN, GD, and JM faced significant regressions.

Indicators *D*_1_ and *D*_2_ reflect the socioeconomic status of a nation and influence the government’s ability to allocate resources for transport safety infrastructure development. The urban population, indicated as *D*_1_, and GDP per capita, denoted as *D*_2_, increased in 29 states, with BS and AG experiencing the most significant growth. In contrast, KN exhibited the most notable decline in these metrics.

Indicators *E*_1_, *E*_2_, *E*_3_, and *E*_4_ focus on evaluating traffic policies and the enforcement of various transport safety laws. Twenty-seven states reported improvements across all four enforcement scores, including speed limit laws, drunk driving laws, seat belt laws, and helmet use laws, with the most significant enhancements observed in AG, UY, and BS. However, LC and GD experienced the most significant declines in *E*_2_, and DM in *E*_3_.

By analyzing changes in overall achievement, organizations and decision-makers can gain insights into the underlying factors driving these changes, prioritize areas for improvement or intervention, and make informed decisions to optimize outcomes.

### Cross-country benchmarking within groups

**Fig. 12:**
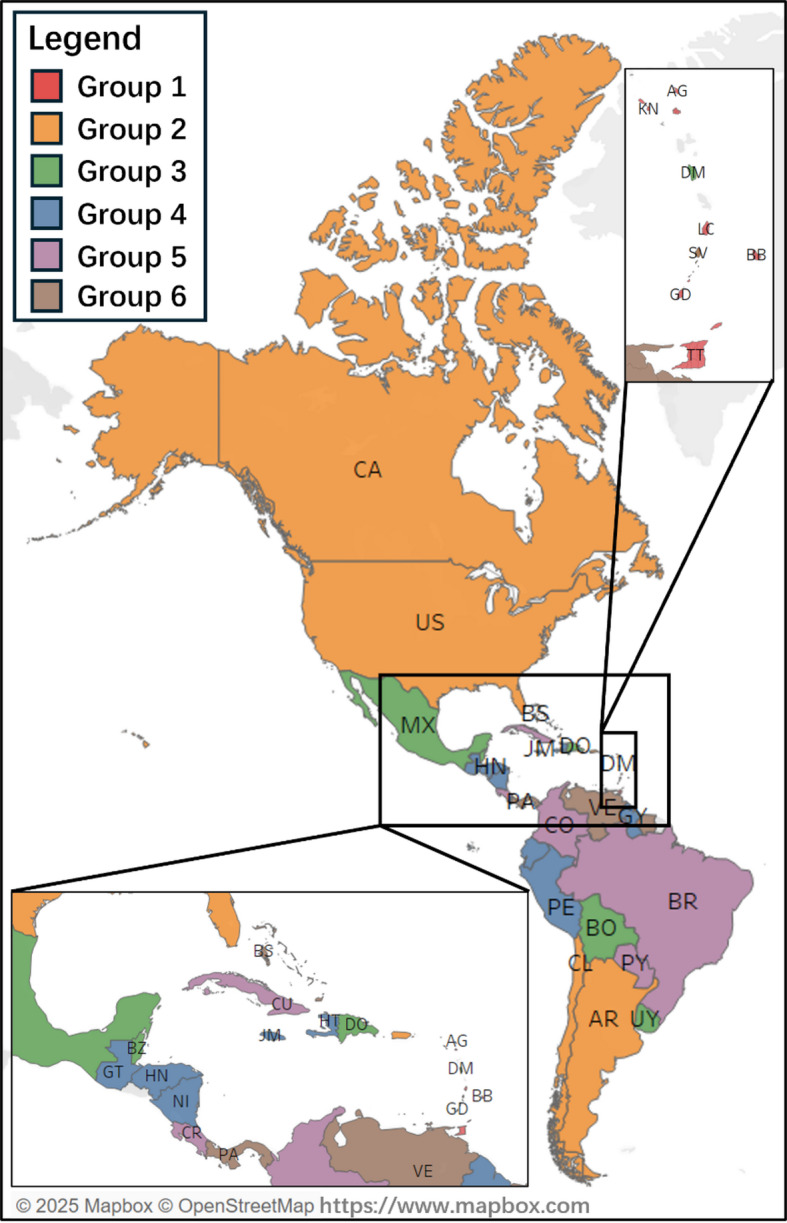
Geographical location of the OAS countries concerning grouping.

**Fig. 13:**
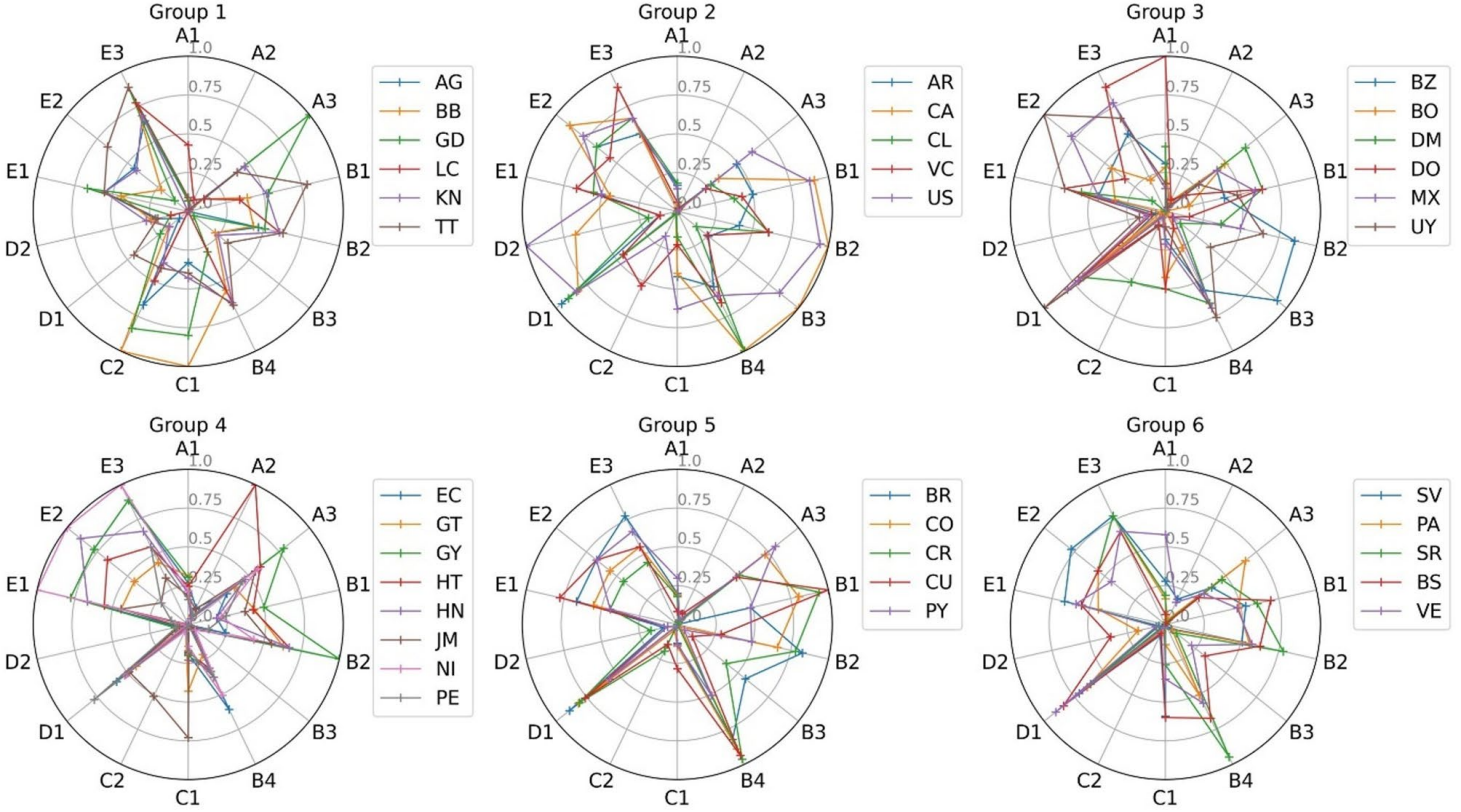
International benchmarking within each group.

To enhance knowledge sharing, collaboration, and continuous improvement within a group, it is crucial to conduct a comprehensive benchmarking of transport performance using specific indicators. This process promotes peer learning, adoption of best practices, and initiatives aimed at enhancing performance, thereby improving the overall performance of the group. Figures 12 and 13 illustrate the grouping of countries based on geographical distribution and the benchmarking of SPIs within each group, respectively.

Figure 13 reveals that a top-ranking country within a group does not necessarily outperform other countries in all indicators. This variation highlights that each country possesses unique strengths and areas needing improvement, offering opportunities for mutual learning and information sharing.

In Group 1, Grenada (GD) shows a particularly strong score on indicator A3, while Barbados (BB) excels in C1 and C2. Trinidad and Tobago (TT) demonstrates notable strengths in E2 and E3, suggesting relative superiority in those aspects compared to its peers. Meanwhile, Saint Kitts and Nevis (KN) displays relatively consistent but moderate values across most indicators, without extreme highs or lows.

In Group 2, Canada (CA) and the United States (US) exhibit high scores in several categories, notably in behavioral and policy-related indicators such as B1 through B4, suggesting strong institutional frameworks or enforcement practices. Saint Vincent and the Grenadines (VC) stands out with top values in E3, indicating notable strengths in that dimension. In contrast, Chile (CL) and Argentina (AR) show more modest or varied performance across the indicators.

In Group 3, the Dominican Republic (DO) stands out with the highest score in A1 and a strong showing in E3, while Uruguay (UY) performs well in indicators E2, D1, and E1, suggesting relative strength in enforcement and institutional factors. Belize (BZ) shows elevated performance in B2 and B3, reflecting strengths in behavioral aspects. In contrast, Bolivia (BO) consistently shows lower values across most indicators, indicating areas for improvement. Mexico (MX) demonstrates relatively balanced outcomes with moderate values across several criteria, and Dominica (DM) shows peaks in A3 and B1.

In Group 4, Haiti (HT) demonstrates high values in A2 and E3, while Guyana (GY) performs strongly in A3 and B2, indicating strengths in enforcement and regulatory domains. Honduras (HN) and Nicaragua (NI) show elevated scores in E1 and E2, suggesting relatively strong institutional or operational aspects. In contrast, Guatemala (GT) and Peru (PE) exhibit more modest or dispersed performances across the indicators. Jamaica (JM) shows some strength in C1 and D1 but underperforms elsewhere.

In Group 5, Cuba (CU) shows high values in behavioral indicators such as B1 and B4, as well as strong scores in E1, suggesting effective safety practices and enforcement. Brazil (BR) stands out in E2 and D1, indicating robust institutional or infrastructural performance. Costa Rica (CR) and Colombia (CO) perform relatively well across multiple indicators, with Colombia showing higher values in A3 and B1. Paraguay (PY), while generally moderate, demonstrates consistent performance without extreme highs or lows.

In Group 6, Suriname (SR) exhibits high values in E2 and B1–B2, suggesting strengths in behavioral compliance and institutional support. El Salvador (SV) performs relatively well in E1 and E2, indicating robust enforcement mechanisms. Panama (PA) stands out in A3 and B1, reflecting strengths in strategic planning and behavior-related indicators. The Bahamas (BS) shows modest performance overall, with peaks in E1 and B4, while Venezuela (VE) scores highly in D1 and E3 but displays more variability across other indicators.

By conducting cross-country benchmarking within groups, stakeholders can obtain valuable insights into relative performance, identify strengths and weaknesses, promote knowledge exchange, and drive continuous improvement efforts among countries facing similar challenges and characteristics, supporting evidence-based policy refinement.

## Concluding remarks

### Conclusions

This study introduces an advanced hybrid MCDM model incorporating a machine learning algorithm, specifically the DCRITIC–WASPAS–K-means model enhanced by a graph-based technique. The proposed model aims to provide a decision-making and policymaking support tool characterized by significant efficiency, stability, and reliability. Through a real case study on transport safety engineering in OAS countries, multiple empirical comparisons highlight the robustness of the proposed model, affirming its practicality, applicability, and adaptability for real-world MCDM tasks. Through the prioritization and classification of countries based on their overall transport safety achievements, the proposed model highlights those that have achieved notable advancements over the past ten years. The dynamic evaluation reveals specific factors contributing to national performance, pinpointing areas where certain countries have excelled. This in-depth assessment equips policymakers with valuable insights into the underlying causes of progress or decline, facilitating a comprehensive review of current strategies. In addition, the benchmarking component delivers tailored policy recommendations for lower-performing nations, enabling them to learn from the experiences and successful practices of peer countries with similar socio-economic conditions. This focused methodology strengthens the contextual relevance and practical value of policy revisions, thereby enhancing the overall impact and efficiency of transport safety programs.

The proposed model is computationally efficient for small to medium-sized datasets. DCRITIC and WASPAS involve low-order operations, while the graph-based enhancement introduces higher complexity—O(n²) for similarity matrix construction and up to O(n³) for eigenvector computation. Although this may limit scalability for large datasets, practical solutions such as sparse graph construction, approximate spectral methods, and parallel processing can significantly reduce runtime. Moreover, the model’s modular design allows for substituting scalable clustering techniques (e.g., mini-batch k-means) without altering its core structure. These adaptations enhance the model’s applicability to larger-scale decision-making contexts while maintaining robustness and interpretability.

This study significantly contributes to both academic research and industry by integrating mathematical models and embedding machine learning algorithms within the MCDM framework.

- First, the composite index developed and analyzed, which includes a set of SPIs, provides a foundational framework adaptable for global benchmarking of transport safety engineering. This framework facilitates a comprehensive view of transport safety achievements and enhances comprehension across different regions.
- Second, this study develops an advanced machine learning embedded MCDM framework for transport safety engineering. It overcomes the traditional limitations of the standard k-means algorithm by incorporating structural relationships among data points through graph theory. Instead of relying solely on Euclidean distance for clustering, this enhanced method constructs a similarity graph—a k-nearest neighbor graph—where nodes represent data points and edges capture meaningful relationships based on proximity, shared attributes, or system interactions. This graph serves as a refined input that guides the clustering process, enabling the algorithm to recognize complex, non-spherical, and irregular cluster structures that reflect the true topology of the data. Additionally, graph-based initialization using centrality measures leads to more stable and representative centroid selection, improving convergence and reducing sensitivity to initial conditions. Unlike traditional initialization methods—such as random seeding, k-means++, or other heuristic approaches—that often suffer from instability, sensitivity to outliers, or poor performance on non-spherical data, our method constructs a similarity graph and derives the Laplacian matrix to capture the intrinsic structure of the dataset. This innovation is particularly valuable in safety engineering reliability contexts, where data often contains interdependent features and represents components within highly structured, interconnected systems.
- Third, the model provides the OAS secretariat and its member countries with a robust diagnostic and decision-making tool to continuously improve transport safety. The empirical findings offer an in-depth evaluation of the transport safety landscape in OAS countries, highlighting key strengths and areas requiring improvement. This detailed understanding aids in the development of precise policies that address specific challenges while capitalizing on existing strengths, thereby facilitating informed policy implementation.

### Limitations and future studies

While the proposed DCRITIC–WASPAS–K-means model integrated with a graph-based technique demonstrates notable improvements in stability, reliability, and applicability within transport safety engineering, several limitations should be acknowledged. First, this study exclusively employed objective weighting techniques (i.e., DCRITIC, Entropy, and MEREC) to derive criteria weights. Although these methods ensure consistency and data-driven transparency, they do not incorporate expert judgment or stakeholder preferences. Subjective or hybrid weighting approaches (e.g., AHP, Delphi, BWM) could capture contextual priorities and policy nuances that objective methods may overlook. Second, the study focused on a specific regional context—OAS countries—and relied on secondary data sources with varying completeness and consistency. The generalizability of the proposed model to other regions or domains (e.g., healthcare, infrastructure, environmental risk) requires further validation. Third, the clustering component of the model, while enhanced through a graph-based initialization technique, assumes a fixed number of clusters (k) and still relies on k-means’ inherent structure. Although spectral properties from the graph provide improved centroids, the model might still face limitations when dealing with overlapping, non-convex, or highly imbalanced clusters.

This study suggests some avenues for further studies. First, future research could explore the integration of hybrid subjective–objective weighting techniques to incorporate expert judgment and contextual priorities into the decision framework, enhancing its adaptability across diverse policy settings. Second, expanding the model’s application to other domains—such as healthcare, environmental risk, or infrastructure resilience—would test its generalizability and further validate its methodological robustness in varied decision-making contexts. Third, future studies may refine the clustering component by incorporating non-parametric or deep learning-based clustering algorithms that better capture overlapping or irregular group structures, particularly in large-scale or high-dimensional datasets where conventional methods face limitations.

## Data Availability

Data will be made available upon reasonable request from the corresponding author.
